# Investigating the relationship between peak inspiratory flow rate and volume of inhalation from a Diskus™ Inhaler and baseline spirometric parameters: a cross-sectional study

**DOI:** 10.1186/2193-1801-3-496

**Published:** 2014-09-02

**Authors:** Jansen N Seheult, Simon Costello, Kee Chun Tee, Tariq Bholah, Hasan Al Bannai, Imran Sulaiman, Richard W Costello

**Affiliations:** Department of Medicine Respiratory Research Division, RCSI-Beaumont Hospital, Beaumont Road, Dublin, Ireland; School of Mathematics, Trinity College Dublin, Dublin, Ireland

**Keywords:** Inhaler technique, Dry powder inhaler, Aerosol delivery, Peak inspiratory flow rate

## Abstract

Drug delivery from a Dry Powder Inhaler (DPI) is dependent on the peak inspiratory flow rate (PIFR) generated. Currently available methods for estimating PIFR from most DPIs are limited and mainly rely on subjective assessment. We aim to show that spirometric and Diskus™ PIFR and Inspiratory Vital Capacity (IVC) are related to the underlying respiratory condition and that spirometric PIFR can be used to assess whether Diskus™ PIFR will be adequate when using this DPI. Healthy volunteers and patients with asthma, COPD, neuromuscular disease and non-respiratory disorders were recruited (n = 85). Demographics and baseline lung function by spirometry were recorded. Flow and volume readings were taken while patients used a Diskus™ DPI, housed in an airtight container connected to a spirometer. T-tests were performed to compare mean spirometric and Diskus™ PIFR/ IVC between groups. Stepwise regression analysis of Diskus™ PIFR versus spirometric PIFR, spirometric IVC, age, gender, condition, BMI, FEV1 and FVC was performed.

The Diskus™ PIFR for the COPD and Neuromuscular Disease group was more than 10 L/min lower than the Healthy or Asthma groups (p < 0.05). The mean spirometric and Diskus™ IVC of the Healthy group was significantly (>0.75 L) higher than the mean for the other three groups (p < 0.05). Diskus™ PIFR was moderately correlated with spirometric PIFR and age (Adjusted R^2^ = 0.58, p < 0.0001). PIFR generated using a Diskus™ DPI is dependent on the underlying disease and age. A spirometric PIFR of less than 196 L/min should prompt further investigation into the suitability of a patient for a Diskus™ DPI, with possible consideration of alternate devices.

**Electronic supplementary material:**

The online version of this article (doi:10.1186/2193-1801-3-496) contains supplementary material, which is available to authorized users.

## Introduction

Inhaled bronchodilators and steroids have been the mainstay of treatment of obstructive airways disease for at least 20 years (Hanania et al. 2012). There has also been a move away from metered dose inhalers (MDIs) towards dry powder inhalers (DPIs) to address the problem of poor coordination that patients face with MDI use. Drug delivery from a DPI is largely dependent on the flow rate generated by the individual (Martonen & Katz 1993). These devices usually have higher resistances that MDIs and a flow rate greater than 60 L/min is necessary to de-aggregate the drug formulation into small particles that can travel to the smaller airways (Burnell et al. 2001; Ganderton 1997),. Therefore, patients’ ability to generate a sufficient peak inhalation flow rate (PIFR) through the device to overcome its resistance is a critical component of a successful inhaler technique for most DPIs.

Janssens et al. (Janssens et al. 2008) and Jarvis et al. (Jarvis et al. 2007) showed that flow rate was very dependent on age in COPD patients; in elderly patients, the ability to generate sufficient inspiratory flow across a dry powder inhaler is compromised, irrespective of the presence of chronic obstructive pulmonary disease. Plavec et al. investigated the proportion of patients with asthma or chronic obstructive pulmonary disease (COPD) with significant broncho-obstruction who do not have inspiratory flows necessary for the adequate use of dry powder inhaler (DPI) devices Diskus™ and Turbuhaler. They found that significant proportions of patients with both asthma and COPD failed to achieve an appropriate flow rate for drug delivery and the proportion of patients who could not generate a high flow rate increased during exacerbations (Plavec et al. 2012). Another study done by Al-Showair et al. also found that PIF from a Diskus™ inhaler was affected by the severity of COPD; however, there were small but significant improvements in the PIF achieved by some patients after training (Al-Showair et al. 2007).

Identification of patients who are likely to achieve a suboptimal PIFR from any DPI is important as a low PIFR can reduce the Fine Particle Dose delivered by more than 50%. Currently, the only available method of determining PIFR from an inhaler is to use a surrogate measure of flow through a Clement-Clarke In-Check Dial. Some studies have used the Clement- Clarke In-Check Dial to study groups of patients with COPD and asthma. This device mimics the resitance of the common DPIs and MDIs and is a surrogate measure of the flow rate an individual achieves from an inhaler (Chrystyn 2003).

One such study compared the accuracy of the In- Check Dial versus Inhalation Profile Recordings from the Diskus™ inhaler in a group of patients with asthma and COPD; the difference between Diskus™_In Check and PIF_ Diskus™ was 3.9 (11.9) L/min (Broeders et al. 2003). The margin of error around the absolute difference is relatively large. Furthermore, not all clinicians have ready access to an In-Check Dial, especially in underdeveloped countries.

An alternative approach is to use lung function measures obtained from baseline spirometry to guide decisions about starting or stopping a DPI. Most patients with obstructive airways disease will have lung function tests performed at least once during the course of their disease. Some authors claim that there is a poor correlation between Diskus™ PIFR and baseline lung function. Plavec et al. concluded that spirometry is not to be used for selection of the device for drug application and In-check Dial should be used instead (Plavec et al. 2012). However, these studies only investigated the correlation between Diskus™ PIFR and FEV1 or PEFR.

Intuitively, there should be a relationship between spirometric PIFR and Diskus™ PIFR_._ This relationship has not been explored in the prior research. In this study, we hypothesized that there is a linear relationship between spirometric PIFR and Diskus™ PIFR_._ We also hypothesized that the mean spirometric and Diskus™ PIFR from a group of patients with COPD or Neuromuscular disease would be significantly lower than healthy volunteers or patients with asthma. Finally, we hypothesized that the mean spirometric and Diskus™ IVC from patients with Asthma, COPD and Neuromuscular Disease would be lower than healthy subjects or patients with non-respiratory disorders.

## Methods

Eighty-five subjects from a population of healthy volunteers and patients with asthma, COPD, neuromuscular disease and non-respiratory disorders were recruited by clustered and stratified sampling. Patients were recruited from different clinics in Beaumont Hospital in Dublin, Ireland. There were no specific exclusion criteria for this study apart from capacity to comply with instructions. Informed consent was obtained for the study with explanations of the study protocol.

This study was approved by the local Hospital Ethics Committee (ERC/ IRB 13/36) and was performed in accordance with the ethical standards laid down in the 2000 Declaration of Helsinki. All participants gave their informed consent prior to their inclusion in the study.

Demographics and baseline lung function by spirometry were recorded (Table 1). Baseline lung function was taken as the best of three trials. Documented parameters included Forced expiratory volume in 1 second (FEV1), Forced Vital Capacity (FVC), FEV1/FVC, Forced inspiratory vital capacity (FIVC) and Peak inspiratory flow rate (PIFR).

**Figure 1:**
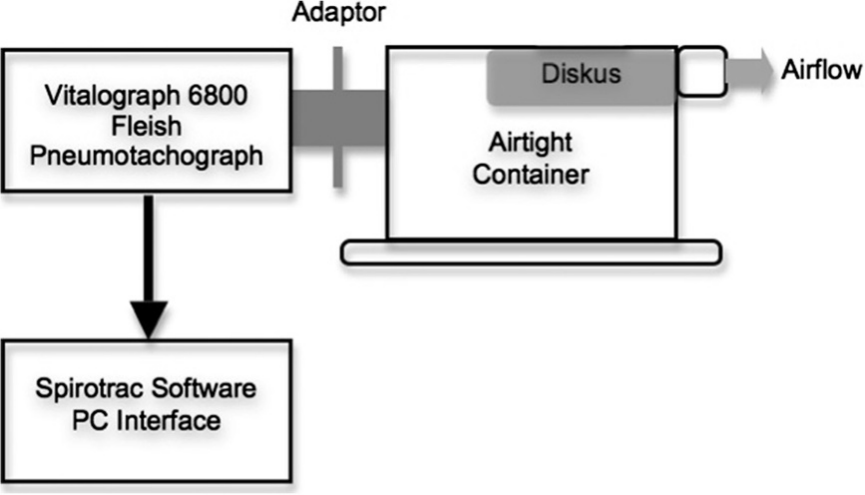
**Apparatus used to measure PIFR and IVC from Diskus™** **inhaler.** Inhaler is sealed inside airtight container with an aperture for connection to a Fleisch pneumotachograph spirometer.

Flow and volume readings were taken while patients used a Diskus™ DPI. The construction of the airtight container with the Diskus™ inhaler, spirometer connection and Fleish Pneumotachograph 6800 spirometer used in this study are shown in Figure 1. Patients were instructed to exhale gently to functional residual capacity and then inhale at maximal flow rate and duration. Each patient performed this manoeuvre until two consecutive PIFR readings were within 20% of each other. Values for Inspiratory vital capacity and peak inspiratory flow rate from the Diskus™ were obtained.

**Table 1:** **Mean values for spirometric and Diskus** ^**TM**^ **PIFR or IVC according to patient disease group**

	Diskus™ PIFR	Spirometric PIFR	Diskus™ IVC	Spirometric IVC
**Healthy/ Non-respiratory condition**	64.57 ± 25.12	247.87 ± 104.35	2.69 ± 1.24	3.37 ± 1.16
	(20–97)	(59–456)	(0.27-4.92)	(1.02-5.42)
**Asthma**	61.56 ± 22.15	209.41 ± 83.26	1.94 ± 0.70	2.42 ± 0.73
	(17–102)	(59–415)	(0.60-3.37)	(1.17-3.78)
**COPD**	49.37 ± 15.68	143.46 ± 62.98	1.86 ± 0.80	2.13 ± 0.79
	(22–83)	(55–275)	(0.36-3.34)	(0.71-3.59)
**Neuromuscular disease**	41.83 ± 24.03	97.53 ± 33.54	1.24 ± 1.13	1.46 ± 1.19
	(10–88)	(55–153)	(0.33-3.87)	(0.42-4.13)

Statistical analysis was done using Audacity v2.0.5., MATLAB v9 and IBM SPSS v21. Ordinary least squares regression of spirometric PIFR versus Diskus™ PIFR was performed to determine the degree of correlation between baseline spirometry and flow or volume inhaled while using the Diskus™ DPI. A stepwise deletion linear regression was also performed to determine the relationship between Diskus™ PIFR and the independent variables: condition (categorical), age, gender (categorical), height, weight, BMI, FEV_1_, FVC, FEV_1_/FVC, spirometric PIFR and spirometric IVC with a significance level for removal from the model of 0.05.

Subjects were classified into subgroups of Healthy/ Non-respiratory illness, Asthma, COPD/ Alpha-1-antitrypsin deficiency, and Neuromuscular Disease. Subgroup analyses were performed for baseline spirometry and Diskus™ spirometry. Multiple t-tests were done to compare the means for spirometric PIFR, Diskus™ PIFR, spirometric IVC and Diskus™ IC for each group. The proportion of patients in each category with a flow rate < 60 L/min and/ or an inspiratory volume < 1 L was also compared. Patients of age greater than or equal to fifty years old and less than fifty years were also compared in the same way.

## Results

**Figure 2:**
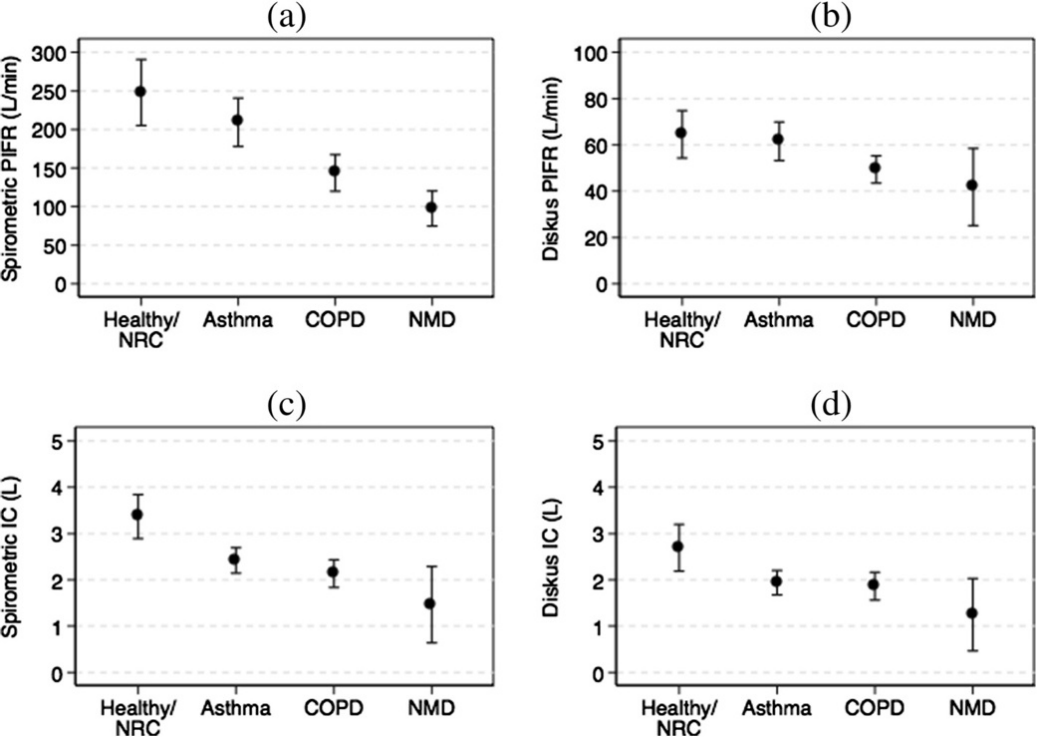
**Mean and 95% Confidence Interval plots for (a) spirometric PIFR versus patient disease group; (b) Diskus™** **PIFR versus patient disease group; (c) spirometric IVC versus patient disease group; and (d) Diskus** ^**TM**^ **IVC versus patient disease group.** NRC: Non-respiratory condition, NMD: Neuromuscular disease.

Approximately two thirds of our recruited patients had a diagnosis of obstructive airways disease (32% asthma and 32% COPD). Twenty seven percent were either healthy or had a non-respiratory condition. Additional file 1: Table S1 shows the demographics and baseline lung function parameters for the subjects and Table 1 shows the mean, standard deviation and range for the spirometric and Diskus™ PIFR and IVC by disease group. Figure 2 shows the mean spirometric PIFR and 95% confidence interval for the 4 groups of patients studied. The p values obtained from the one-sided independent samples t-test for the mean differences between groups are shown in Table 2(a). The mean spirometric PIFR was significantly lower for the COPD and Neuromuscular disease groups compared to the Asthma or Healthy/ Non-respiratory condition groups (p ≤ 0.001). These trends were also seen for mean Diskus™ PIFR values (Figure 2(b) and Table 2). Additionally, the mean spirometric PIFR for the Neuromuscular Disease group was significantly lower than that for the COPD group (p = 0.005). The Diskus™ PIFR for the COPD and Neuromuscular Disease groups was more than 10 L/min lower than the Healthy or Asthma groups and was significantly lower than 60 L/min (p < 0.05). The proportions of patients in each group with a Diskus™ PIFR < 60 L/min (the threshold for optimum drug delivery from the Diskus™ inhaler) were significantly different (Additional file 1: Table S2).

**Table 2:** **p values for one-sided independent samples t-tests for comparisons of spirometric and Diskus™** **PIFR and IVC between patient disease groups**

	Peak Inspiratory Flow Rate (PIFR)			
	Healthy/Non-respiratory condition	Asthma	COPD	Neuromuscular disease
**Healthy/Non-respiratory condition**	--	NS *(d)*	0.009 *(d)*	0.019 *(d)*
**Asthma**	NS *(s)*	--	0.012 *(d)*	0.030 *(d)*
**COPD**	0.000 *(s)*	0.001 *(s)*	--	NS *(d)*
**Neuromuscular disease**	0.000 *(s)*	0.000 *(s)*	0.005 *(s)*	--
	**Inspiratory Vital Capacity (IVC)**			
	**Healthy/Non-respiratory condition**	**Asthma**	**COPD**	**Neuromuscular disease**
**Healthy/Non-respiratory condition**	--	0.004 *(d)*	0.004 *(d)*	0.004 *(d)*
**Asthma**	0.001 *(s)*	--	NS *(d)*	NS *(d)*
**COPD**	0.000 *(s)*	NS *(s)*	--	NS *(d)*
**Neuromuscular disease**	0.001 *(s)*	0.030 *(s)*	NS *(s)*	--

(s) represensts spirometric values and (d) represents Diskus^TM^ values. NS: not significant at an alpha of 0.05.

The mean spirometric and Diskus™ IVC values with 95% CIs and the p-values for differences between group means are shown in Figures 2(c) and (d) and Tables 1 and 2. The mean spirometric and Diskus™ IVC of the Healthy group was significantly (>0.75 L) higher than the mean for the other three groups (p ≤ 0.001 for spirometric PIFR; p = 0.004 for Diskus™ PIFR). The spirometric IVC was also higher for the Asthma and COPD groups compared to the Neuromuscular Disease group; the differences were not statistically significant except for the spirometric IVC from the Asthma group compared to the Neuromuscular Disease group (p = 0.03).

The p-values for two-sided independent samples t-tests for age groups, BMI groups and Gender groups are shown in Table 3. The mean values for all four measured flow rate and volume parameters were significantly higher in younger patients (age <50 years). There was no difference between the obese (BMI >30 kg/m^2^) versus non-obese groups nor the male versus female groups.

**Table 3:** **Mean differences and p values for two-sided independent samples t-tests for comparisons of spirometric**

	Age (<50 vs > =50)	Male vs Female	BMI (<30 vs > =30)
**Diskus PIFR**	9.61 (0.025)	−0.429 (NS)	3.609 (NS)
**Baseline PIFR**	48.33 (0.017)	23.543 (NS)	−2.082 (NS)
**Diskus IVC**	0.4803 (0.030)	0.2187 (NS)	0.1492 (NS)
**Baseline IVC**	0.7755 (0.003)	0.8289 (NS)	0.0646 (NS)

The stepwise deletion regression showed that gender, height, weight, BMI, FEV_1_, FVC and FEV_1_/FVC were not significantly correlated with Diskus™ PIFR at a significance level of 0.05. Diskus™ PIFR was moderately correlated with spirometric PIFR and age (adjusted R^2^ = 0.58, p < 0.0001) and the relationship is described by the following equation:
1

**Figure 3:**
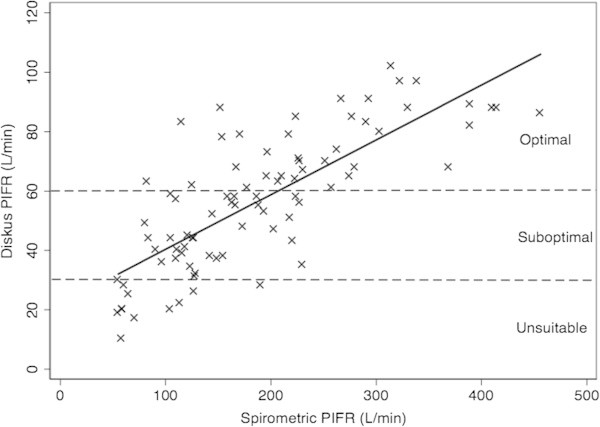
**Scatterplot of Diskus™** **PIFR versus spirometric PIFR showing line of best fit.** Dashed lines represent Diskus PIFR of 30 L/min (minimum required) and 60 L/min (optimal PIFR for drug delivery). Patients with a Diskus PIFR less than 30 L/min are unsuitable for this dry powder inhaler.

While Diskus™ PIFR from COPD and Neuromuscular disease patients was significantly different to that from healthy patients and asthmatics, this effect was modified by both age and spirometric PIFR in the stepwise regression and as a result, condition was no longer significant in the model. A scatterplot of Diskus™ PIFR versus spirometric PIFR with the line of best fit are shown in Figure 3.

Diskus™ PIFR was binned according to a threshold of 60 L/min and a ROC Curve of Spirometric PIFR versus Diskus™ PIFR category had an area under the curve of 0.89. At a spirometric PIFR cutoff of 196 L/min, 84% of Diskus™ PIFR values were correctly classified as either greater than or equal to 60 L/min or less than 60 L/min (sensitivity of 79% and specificity of 87%). When the Diskus™ PIFR was binned according to a threshold of 30 L/min, a spirometric cutoff of 115 L/min had a sensitivity of 86% and a specificity of 83% (86% correctly classified). The ROC Curves for a Diskus™ threshold of 60 L/min 30 L/min is shown in Additional file 1: Figures S1 and S2.

## Discussion

Our results show that patients with COPD and Neuromuscular Disease do not generate as high a PIFR (spirometric or through Diskus™) as Healthy subjects, Asthmatics or patients with non-respiratory conditions. Healthy subjects also have a significantly higher spirometric and Diskus™ IVC compared to patients with respiratory-related diseases. Numerous authors have shown that drug delivery from a Diskus™ DPI is dependent on the PIFR of inhalation and that ideally, the PIFR should be above 60 L/min for optimum Fine Particle delivery. It is clear that the decision to start a patient on an inhaled medication delivered via a Diskus^TM^ DPI should take into account the age of the patient and the underlying disease. It is likely that the differences seen between the PIFR of asthmatics versus COPD patients is explained by the fact that the COPD patients who were recruited for this study were older than the patients in the other groups. The lower PIFR in the patients with Neuromuscular Disease is most likely explained by the pathophysiology of their underlying disease process leading to poor muscle function and contraction.

The second aim of our study was to show that the baseline spirometric PIFR could be used to estimate whether patients would be suitable for the Diskus™ DPI based on PIFR criteria. There was a moderate correlation between spirometric and Diskus™ PIFR and the use of spirometric PIFR was very sensitive and specific for categorizing the Diskus™ PIFR as either greater than or equal to 60 L/min or less than 60 L/min. The amount of variance in Diskus™ PIFR explained by the spirometric PIFR is much higher than published values for FEV1 or FVC in the literature. The use of subjective evaluation of PIFR from a DPI is obviously sub-optimal due to the high inter- and intra- rater variability. In the absence of a Clement-Clarke In-Check Dial for estimating Diskus™ PIFR, we believe that our method allows a much better estimation of the flow rate of inhalation from the Diskus™ than subjective assessment.

Spirometric PIFR cutoffs of 196 L/min or 115 mL/min correlate with a Diskus™ PIFR of 60 L/min (optimal delivery) and 30 L/min (minimum required for successful use), respectively. Our study shows that no patient with a spirometric PIFR above 196 L/min had a Diskus PIFR below 30 L/min. We believe that the 196 L/min spirometric cutoff is the more useful of the two in the general practice setting. It will identify all patients who are likely to have the minimal required Diskus™ PIFR of 30 L/min. Any patient with a spirometric PIFR below 196 L/min should have further testing possibly using the Clement Clark In-check Dial™ or consideration of an alternate device.

While our study shows that spirometric PIFR can be used to aid decision making regarding suitability of a patient for a Diskus™ DPI, it is likely that the same relationship exists for other dry powder devices that are also flow-rate dependent. However, without studying these devices, we are unable to make any suggestions regarding cutoffs for other DPIs. Each device has a different intrinsic resistance; the Turbohaler™ for instance is even more peak flow dependent than the Diskus™. We plan to study these devices in the future but since it is our local experience that the Diskus is the most popular DPI, we decided to focus this pilot study on this device.

Based on the stepwise deletion regression, it is clear that Diskus™ PIFR is related to both spirometric PIFR and to age. Interestingly, condition was not a significant variable in this model and it is likely that the differences in Diskus™ PIFR seen among diseases is explained by the differences in mean age across the groups. As expected, COPD patients were older than the other groups and had lower PIFRs than asthmatics and healthy patients. Since these three groups were the largest in this study, it is clear why Diskus™ PIFR is also related to age. Age should therefore be taken into account when making a decision about suitability for a Diskus™ DPI.

Our study has a few mentionable limitations. Technical issues related to the rig used to measure Diskus™ PIFR and IVC have been documented previously (Holmes et al. 2013). More importantly, our method for estimation of Diskus™ PIFR is not perfect (we could not explain about 40% of the variance seen in Diskus™ PIFR by using spirometric PIFR). PIFR is a very effort-dependent measure and variations in effort exerted by the patient could explain the differences seen in spirometric and Diskus™ PIFR values. Finally, the need to assess PIFR from a DPI is important for traditional DPIs for which drug delivery is flow-rate dependent but may be unnecessary in the future when the use of modern, sophistically engineered dry powder formulations and devices becomes more widespread.

Patients with COPD and Neuromuscular Disease may not obtain the same drug delivery from a Diskus™ DPI compared to patients with Asthma and inhaler technique training for these groups needs to focus on the flow rate generated during inhaler use. We propose the use of spirometric PIFR to estimate the PIFR generated during use of the Diskus™ inhaler in order to guide device selection and technique training.

## Conclusion

Patients with COPD and Neuromuscular Disease have a lower spirometric PIFR and PIFR from a Diskus™ DPI than healthy subjects or patients with Asthma. Measurement of spirometric PIFR can be used as a surrogate to estimate the PIFR a patient is likely to generate while using the Diskus™ DPI. A spirometric PIFR of less than 196 L/min should prompt further investigation into the suitability of a patient for a Diskus™ DPI, with possible consideration of alternate devices. Patient age should also be considered when selecting this common dry powder inhaler.

## Electronic supplementary material

Additional file 1: Table S1: Demographics and baseline lung function tests for patients by disease category. **Table S2.** Number of patients in each disease group with a Diskus^TM^ PIFR greater than or equal to 60 L/min and less than 60 L/min. Results from Chi-squared test are shown. The null hypothesis is that the proportions of patients with a Diskus™ PIF value less than or equal to sixty is independent of their diagnosis.As the test statistic is greater than the critical value, we can reject the null hypothesis. **Figure S1.** Receiver Operating Characteristic Curve for spirometric PIFR versus binary Diskus™ PIFR based on threshold of 60 L/min. The solid line represents an AUC of 0.5. **Figure S2.** Receiver Operating Characteristic Curve for spirometric PIFR versus binary Diskus™ PIFR based on threshold of 30 L/min. The solid line represents an AUC of 0.5. (DOCX 232 KB)

## References

[CR1] Al-Showair RA, Tarsin WY, Assi KH, Pearson SB, Chrystyn H (2007). Can all patients with COPD use the correct inhalation flow with all inhalers and does training help?. Respir Med.

[CR2] Broeders ME, Molema J, Vermue NA, Folgering HT (2003). In check dial: accuracy for Diskus and Turbuhaler. Int J Pharm.

[CR3] Burnell PK, Small T, Doig S, Johal B, Jenkins R, Gibson GJ (2001). Ex-vivo product performance of DiskusTM and Turbuhaler inhalers using inhalation profiles from patients with severe chronic obstructive pulmonary disease. Respir Med.

[CR4] Chrystyn H (2003). Is inhalation rate important for dry powder inhalers? Using the In-Check Dial to identify these rates. Respir Med.

[CR5] Ganderton D (1997). General factors inflencing drug delivery to the lung. Respir Med.

[CR6] Hanania NA, Crater GD, Morris AN, Emmett AH, O’Dell DM, Niewoehner DE (2012). Benefits of adding fluticasone propionate/salmeterol to tiotropium in moderate to severe COPD. Respir Med.

[CR7] Holmes MS, Seheult JN, Geraghty C, D’Arcy S, O’Brien U, Crispino O’Connell G, Costello RW, Reilly RB (2013). A method of estimating inspiratory flow rate and volume from an inhaler using acoustic measurements. Physiol Meas.

[CR8] Janssens W, VandenBrande P, Hardeman E, De Langhe E, Philps T, Troosters T, Decramer M (2008). Inspiratory flow rates at different levels of resistance in elderly COPD patients. Eur Respir J.

[CR9] Jarvis S, Ind PW, Shiner RJ (2007). Inhaled therapy in elderly COPD patients; time for re-evaluation?. Age Ageing.

[CR10] Martonen B, Katz IM (1993). Deposition patterns of aerosolised drugs within human lungs. Effects of ventilatory parameters. Pharm Res.

[CR11] Plavec D, Gluncić TJ, Gudelj I, Mise K (2012). Measurement of inspiratory flow for the selection of the inhalation treatment device for asthma and COPD. Lijec Vjesn.

