# Supplementary material for: Investigating the relationship between peak inspiratory flow rate and volume of inhalation from a Diskus™ Inhaler and baseline spirometric parameters: a cross-sectional study
Source: Springerplus. 2014 Sep 2;3:496. doi: 10.1186/2193-1801-3-496 (PMC4164676; doi:10.1186/2193-1801-3-496)
Supplement: Supplementary file 1 — Additional file 1: Table S1: Demographics and baseline lung function tests for patients by disease category. Table S2. Number of patients in each disease group with a DiskusTM PIFR greater than or equal to 60 L/min and less than 60 L/min. Results from Chi-squared test are shown. The null hypothesis is that the proportions of patients with a Diskus™ PIF value less than or equal to sixty is independent of their diagnosis.As the test statistic is greater than the critical value, we can reject the null hypothesis. Figure S1. Receiver Operating Characteristic Curve for spirometric PIFR versus binary Diskus™ PIFR based on threshold of 60 L/min. The solid line represents an AUC of 0.5. Figure S2. Receiver Operating Characteristic Curve for spirometric PIFR versus binary Diskus™ PIFR based on threshold of 30 L/min. The solid line represents an AUC of 0.5. (DOCX 232 KB) [file 40064_2014_1202_MOESM1_ESM.docx]

**Additional file 1**

|  | All | Asthma | COPD | Neuro-muscular Disease | Healthy/ Non-respiratory Condition |
| --- | --- | --- | --- | --- | --- |
| Number | 85 | 27 | 27 | 8 | 23 |
| Age  (years) | 51.8±17.8  (18-80) | 52.6±15.9  (18-76) | 66.0±8.4  (44-80) | 41.9±19.6  (18-78) | 37.8 ± 14.6  (20-65) |
| Gender  (M:F%) | 42:58 | 30:70 | 37:63 | 75:25 | 52:48 |
| BMI  (kg/m^2^) | 27.6±6.8  (16.7-49.2) | 27.0±6.0  (16.7-37.8) | 26.2±5.3  (18.0-38.0) | 29.0±8.6  (21.3-48.2) | 29.5±8.6  (19.5-49.2) |
| FEV_1_  (L) | 2.02±1.04  (0.24-5.07) | 1.74±0.70  (0.93-3.38) | 1.56±0.75  (0.24-3.05) | 1.29±1.21  (0.33-3.06) | 3.13±0.90  (1.58-5.07) |
| FVC  (L) | 2.75±1.12  (0.38-5.66) | 2.45±0.78  (1.23-3.99) | 2.41±0.79  (0.63-3.60) | 1.69±1.69  (0.38-4.18) | 3.83±1.02  (1.89-5.66) |
| FEV_1_/  FVC | 0.72±0.16  (0.35-0.99) | 0.71±0.16  (0.44-0.94) | 0.62±0.16  (0.35-0.85) | 0.80±0.09  (0.71-0.90) | 0.83±0.08  (0.70-0.99) |

*Table S1: Demographics and baseline lung function tests for patients by disease category.*

*Table S2: Number of patients in each disease group with a Diskus^TM^ PIFR greater than or equal to 60 L/min and less than 60 L/min. Results from Chi-squared test are shown.The null hypothesis is that the proportions of patients with a Diskus^TM^ PIF value less than or equal to sixty is independent of their diagnosis.As the test statistic is greater than the critical value, we can reject the null hypothesis*.

| PIFR (L/min) | Healthy/ Non-respiratory condition | Asthma | COPD | Neuromuscular Disease | Totals |
| --- | --- | --- | --- | --- | --- |
| ≥ 60 | 14 | 15 | 8 | 1 | 38 |
| <60 | 9 | 12 | 19 | 7 | 47 |
| Totals | 23 | 27 | 27 | 8 | 85 |

*Chi-squared Statistic= 8.04; Critical Value= 7.815; df= 3*

*Figure S1: Receiver Operating Characteristic Curve for spirometric PIFR versus binary Diskus^TM^ PIFR based on threshold of 60 L/min. The solid line represents an AUC of 0.5.*


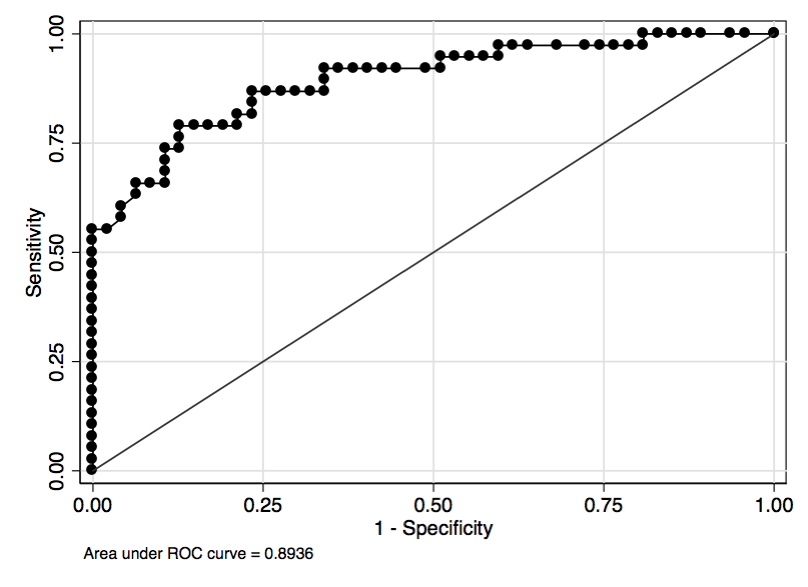


*Figure S2: Receiver Operating Characteristic Curve for spirometric PIFR versus binary Diskus^TM^ PIFR based on threshold of 30 L/min. The solid line represents an AUC of 0.5.*

*
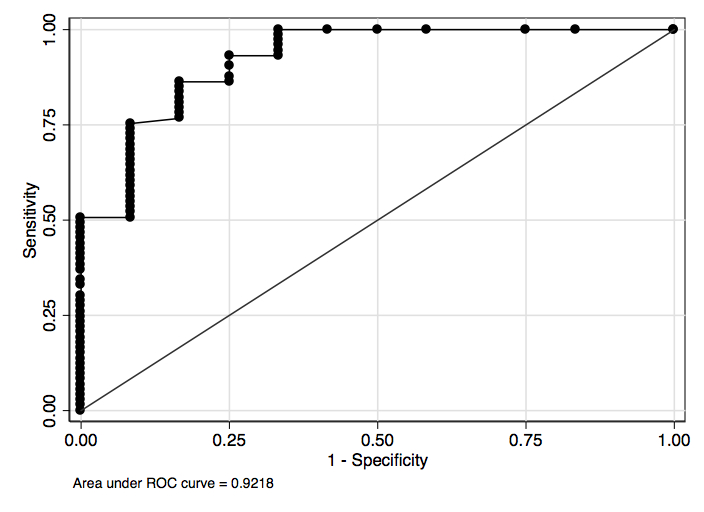
*
